# Crystal structures of 2-[(4,6-di­amino­pyrimidin-2-yl)sulfan­yl]-*N*-(3-nitro­phen­yl)acetamide monohydrate and *N*-(2-chloro­phen­yl)-2-[(4,6-di­amino­pyrimidin-2-yl)sulfan­yl]acetamide

**DOI:** 10.1107/S2056989016011658

**Published:** 2016-07-22

**Authors:** S. Subasri, Ajay Kumar Timiri, Nayan Sinha Barji, Venkatesan Jayaprakash, Viswanathan Vijayan, Devadasan Velmurugan

**Affiliations:** a Centre of Advanced Study in Crystallography and Biophysics, University of Madras, Guindy Campus, Chennai 600 025, India; b Department of Pharmaceutical Sciences, Birla Institute of Technology, Mesra, Ranchi, India

**Keywords:** crystal structure, acetamides, intra­molecular N—H⋯O and N—H⋯Cl hydrogen bonds, network, framework

## Abstract

The title compounds, C_12_H_12_N_6_O_3_S·H_2_O, (I), and C_12_H_12_ClN_5_OS, (II), are 2-[(4,6-di­amino­pyrimidin-2-yl)sulfan­yl]acetamides. Compound (I) crystallized as a monohydrate. In both compounds, the mol­ecules have a folded conformation, with the pyrimidine ring being inclined to the benzene ring by 56.18 (6)° in (I) and by 67.84 (6)° in (II). In both mol­ecules, there is an intra­molecular N—H⋯N hydrogen bond stabilizing the folded conformation. In (I), there is also a C—H⋯O intra­molecular short contact, and in (II) an intra­molecular N—H⋯Cl hydrogen bond is present. In the crystal of (I), mol­ecules are linked by a series of N—H⋯O, O—H⋯O and O—H⋯N hydrogen bonds, forming undulating sheets parallel to the (100). The sheets are linked *via* an N—H⋯O_water_ hydrogen bond, forming a three-dimensional network. In the crystal of (II), mol­ecules are linked by a series of N—H⋯O, N—H⋯N and C—H⋯O hydrogen bonds, forming slabs parallel to (001).

## Abstract 2

In the title 2-[(4,6-di­amino­pyrimidin-2-yl)sulfan­yl]acetamides, both compounds have a folded conformation about the methyl­ene C atom of the thio­acetamide bridge, with the pyrimidine ring being inclined to the benzene ring by 56.18 (6) and 67.84 (6)°. In both mol­ecules, there is an intra­molecular N—H⋯N hydrogen bond stabilizing the folded conformation.

## Chemical context   

Recent studies have shown that di­amino substituted pyrimidines are active inhibitors of human di­hydro­folate reductase (hDHFR) and also possess inhibitory potency against tyrosine kinase (Gangjee *et al.*, 2006 ▸). 2,4-di­amino pyrimidine derivatives have anti-retro viral activity (Hocková *et al.*, 2004 ▸) and also anti-trypanosoma brucei activity (Perales *et al.*, 2011 ▸). A series of 2,4-di­amino­pyrimidines have as also been prepared to study their immuno-suppressant activity (Blumenkopf *et al.*, 2003 ▸). Pyrimidines are also potent anti­viral agents and a series of *N*-benzyl-2-(4,6-di­amino­pyrimidin-2-ylsulfan­yl)acetamides have been designed to fight Dengue Virus Protease (Timiri *et al.*, 2016 ▸). A series 5-substituted benzyl-2,4-di­amino pyrimidine derivatives have also been synthesized as c-Fms kinase inhibitors (Xu *et al.*, 2010 ▸). As part of our studies in this area, we now describe the syntheses and crystal structures of the title compounds.

## Structural commentary   

**Figure 1:**
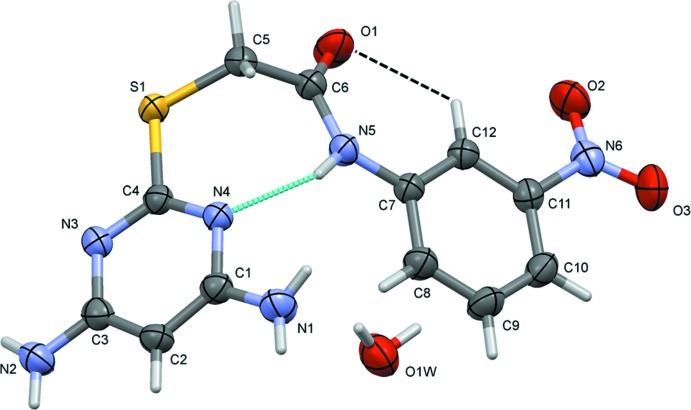
The mol­ecular structure of compound (I), with atom labelling. Displacement ellipsoids are drawn at the 50% probability level. Intra­molecular hydrogen bonds are shown as dashed lines (see Table 1 ▸).

The mol­ecular structures of compounds (I) and (II) are illus­trated in Figs. 1 ▸ and 2 ▸, respectively. In compound (I), the pyrimidine ring makes a dihedral angle of 56.18 (6)° with the benzene ring (C7–C12). The nitro group is inclined by 16.3 (3)° to the benzene ring to which it is attached. The amine nitro­gen atoms, N1 and N2, are displaced from the pyrimidine ring by 0.028 (2) and 0.026 (2) Å, respectively.
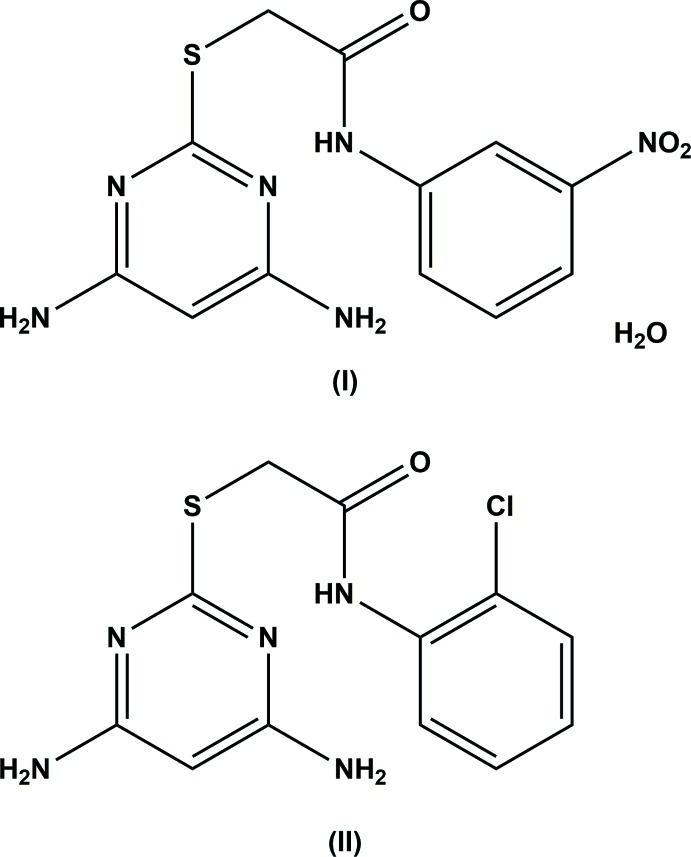


In compound (II), the pyrimidine ring makes a dihedral angle of 67.84 (6)° with the chloro­benzene ring (C7–C12). The amine nitro­gen atoms, N1 and N2, are displaced from the pyrimidine ring by 0.009 (2) and 0.030 (2) Å, respectively. The chlorine atom, Cl1, attached to the benzene ring deviates by 0.053 (1) Å from the ring plane.

**Figure 2:**
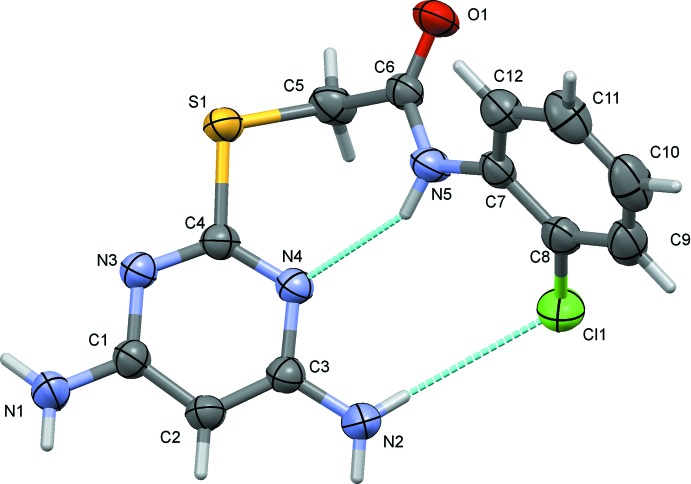
The mol­ecular structure of compound (II), with atom labelling. Displacement ellipsoids are drawn at the 50% probability level. Intra­molecular hydrogen bonds are shown as dashed lines (see Table 2 ▸).

In both the compounds, the folded conformation is reinforced by an intra­molecular N—H⋯O hydrogen bond [Fig. 1 ▸, Table 1 ▸ for (I) and Fig. 2 ▸, Table 2 ▸ for (II)]. In (I) there is an intra­molecular C—H⋯O contact (Table 1 ▸ and Fig. 1 ▸) and in (II) an intra­molecular N—H⋯Cl hydrogen bond is also present (Table 2 ▸ and Fig. 2 ▸).

## Supra­molecular features   

**Figure 3:**
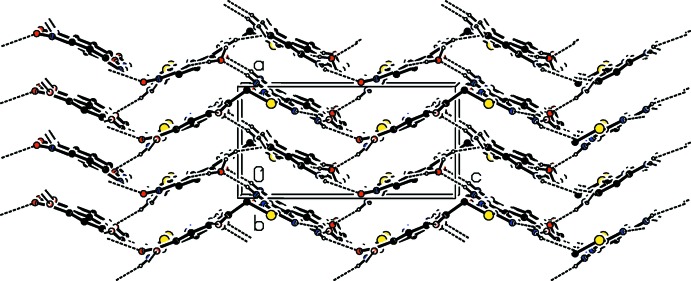
The crystal packing of compound (I), viewed along the *b* axis. Hydrogen bonds are shown as dashed lines (see Table 1 ▸). C-bound H atoms have been excluded for clarity.

**Figure 4:**
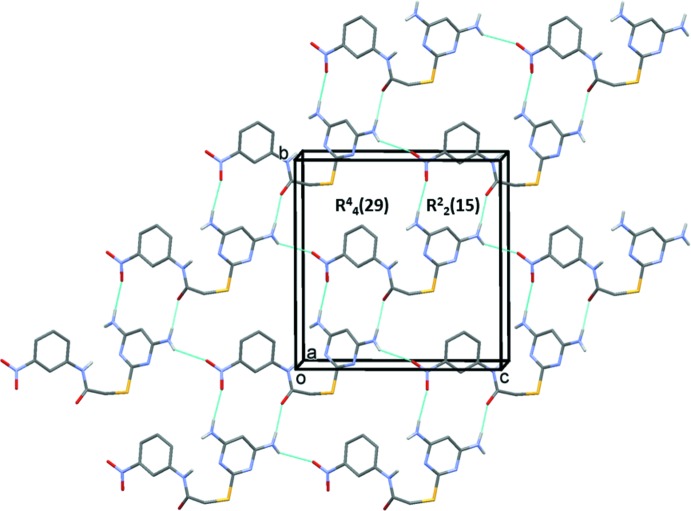
A view of the hydrogen-bonded ring motifs in the crystal of compound (I). Details of the hydrogen bonding are given in Table 1 ▸.

In the crystal of compound (I), mol­ecules are linked by a series of N—H⋯O, O—H⋯O and O—H⋯N hydrogen bonds, forming undulating sheets parallel to the *bc* plane (Table 1 ▸ and Fig. 3 ▸). The sheets are linked *via* an N—H⋯O_water_ hydrogen bond, forming a three-dimensional network (Table 1 ▸ and Fig. 3 ▸). Through pairs of N—H⋯O hydrogen bonds, 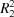
(15) and 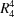
(29) ring motifs are generated (Table 1 ▸ and Fig. 4 ▸).

**Figure 5:**
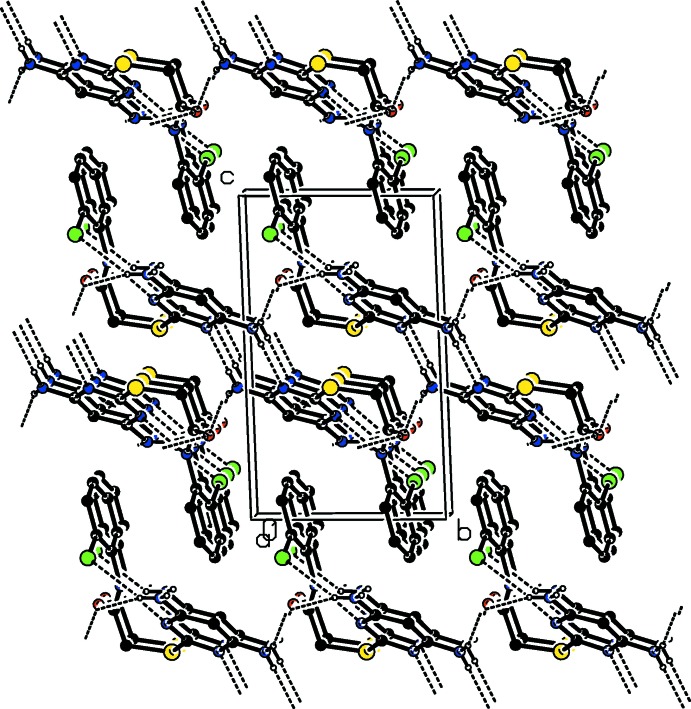
The crystal packing of compound (II), viewed along the *a* axis. Hydrogen bonds are shown as dashed lines (see Table 2 ▸)·C-bound H atoms have been excluded for clarity.

**Figure 6:**
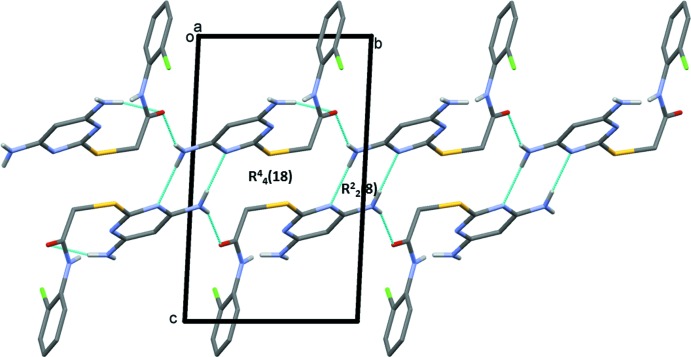
A view of the hydrogen-bonded ring motifs in the crystal of compound (II). Details of the hydrogen bonding are given in Table 2 ▸.

In the crystal of compound (II), mol­ecules are linked by a series of N—H⋯O, N—H⋯N and C—H⋯O hydrogen bonds, forming slabs parallel to the *ab* plane (Table 2 ▸ and Fig. 5 ▸). Through pairs of N—H⋯N hydrogen bonds, *R* ^2^_2(_8) ring motifs are generated, and through further pairs of N—H⋯N and N—H⋯O hydrogen bonds 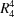
(18) ring motifs are also formed (Table 2 ▸ and Fig. 6 ▸).

## Database survey   

A search of the Cambridge Structural Database (Version 5.37, update May 2016; Groom *et al.*, 2016 ▸) for 2-(pyrimidin-2-ylsulfan­yl)-*N*-phenyl­acetamides yielded only three hits. There are two 4,6-di­methyl­pyrimidine analogues *viz*. 2-(4,6-di­meth­yl­pyrimidin-2-ylsulfan­yl)-*N*-phenyl­acetamide (DIWXAJ; Gao *et al.*, 2008 ▸) and *N*-(2-chloro­phen­yl)-2-(4,6-di­methyl­pyrimidin-2-ylsulfan­yl)acetamide QOTQEW; Li *et al.*, 2009 ▸), but only one 4,6-di­amino­pyrimidine compound *viz*. 2-[(4,6-diamino­pyrimidin-2-yl)sulfan­yl]-*N*-(2-methyl­phen­yl)acetamide (GOKWIO; Subasri *et al.*, 2014 ▸). In the 4,6-di­methyl­pyrimidine analogues, DIWXAJ and QOTQEW, the pyrimidine ring is inclined to the benzene ring by 88.86 (15) and 79.60 (8)°, respectively. In the 4,6-di­amino­pyrimidine compound, GOKWIO, the two rings are inclined to one another by 54.73 (9)°. This last value is similar to that observed in the compound (I), *viz.* 56.18 (6)°.

## Synthesis and crystallization   

**Compound (I**): To a solution of 4,6-di­amino-pyrimidine-2-thiol (0.5 g; 3.52 mmol) in 25 ml of ethanol in a round-bottom flask, potassium hydroxide (0.2 g; 3.52 mmol) was added and the mixture was refluxed for half an hour and to it 3.52 mmol of 3-nitro phenyl­acetamide was added and refluxed for 4 h. At the end of the reaction (observed by TLC), ethanol was evaporated under vacuum and cold water was added and the precipitate filtered and dried to give compound (I) as a crystalline powder (yield 88–96%). After purification, the compound was recrystallized from ethyl acetate solution by slow evaporation of the solvent.

**Compound (II)**: To a solution of 4,6-di­amino-pyrimidine-2-thiol (0.5 g; 3.52 mmol) in 25 ml of ethanol in a round-bottom flask potassium hydroxide (0.2 g; 3.52 mmol) was added and refluxed for half an hour and to it 3.52 mmol of 2-chloro-phenyl­acetamide was added and the mixture was refluxed for 3 h. At the end of the reaction (observed by TLC), ethanol was evaporated under vacuum and cold water was added, and the precipitate was filtered and dried to give compound (II) as a crystalline powder (yield 88–96%). After purification, the compound was recrystallized from ethanol solution by slow evaporation of the solvent.

## Refinement   

Crystal data, data collection and structure refinement details are summarized in Table 3 ▸. For both compounds, the NH_2_ and NH H atoms, and the water H atoms for (I), were located in difference Fourier maps. The N-bound H atoms were freely refined, while the water H atoms were initially freely refined and in the final cycles of refinement as riding atoms. The C-bound H atoms were placed in calculated positions and refined as riding: C—H = 0.93–0.97 Å with *U*_iso_(H) = 1.2*U*_eq_(C).

## Supplementary Material

Crystal structure: contains datablock(s) global, I, II. DOI: 10.1107/S2056989016011658/su5311sup1.cif

Structure factors: contains datablock(s) I. DOI: 10.1107/S2056989016011658/su5311Isup2.hkl

Supporting information file. DOI: 10.1107/S2056989016011658/su5311Isup4.cml

Structure factors: contains datablock(s) II. DOI: 10.1107/S2056989016011658/su5311IIsup3.hkl

Supporting information file. DOI: 10.1107/S2056989016011658/su5311IIsup5.cml

CCDC references: 1494258, 1494257

Additional supporting information:  crystallographic information; 3D view; checkCIF report

## Figures and Tables

**Table 1:** Hydrogen-bond geometry (Å, °) for (I)

*D*—H⋯*A*	*D*—H	H⋯*A*	*D*⋯*A*	*D*—H⋯*A*
N5—H5⋯N4	0.86 (3)	2.05 (3)	2.832 (3)	151 (3)
C12—H12⋯O1	0.93	2.35	2.911 (3)	118
N1—H1*A*⋯O1*W* ^i^	0.83 (3)	2.16 (3)	2.979 (3)	170 (2)
N1—H1*B*⋯O2^ii^	0.84 (3)	2.29 (3)	3.082 (3)	159 (3)
N2—H2*A*⋯O3^iii^	0.80 (3)	2.58 (3)	3.255 (3)	143 (3)
N2—H2*B*⋯O1^ii^	0.83 (3)	2.09 (3)	2.904 (3)	170 (3)
O1*W*—H1*WA*⋯N3^iv^	0.86	2.09	2.919 (3)	162
O1*W*—H1*WB*⋯O3^v^	0.90	2.64	3.294 (3)	130

Symmetry codes: (i) 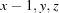; (ii) 
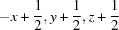
; (iii) 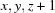; (iv) 
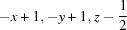
; (v) 
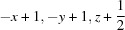
.

**Table 2:** Hydrogen-bond geometry (Å, °) for (II)

*D*—H⋯*A*	*D*—H	H⋯*A*	*D*⋯*A*	*D*—H⋯*A*
N5—H5⋯N4	0.85 (2)	2.12 (2)	2.898 (2)	152 (2)
N2—H2*A*⋯Cl1	0.81 (3)	2.81 (2)	3.493 (2)	143 (2)
N1—H1*A*⋯N3^i^	0.85 (2)	2.21 (2)	3.058 (2)	174 (2)
N1—H1*B*⋯O1^ii^	0.83 (2)	2.21 (2)	2.992 (2)	157 (2)
N2—H2*A*⋯O1^iii^	0.81 (3)	2.56 (2)	3.095 (2)	124 (2)
C2—H2⋯O1^ii^	0.93	2.64	3.353 (2)	134

Symmetry codes: (i) 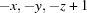; (ii) 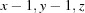; (iii) 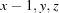.

**Table 3:** Experimental details

	(I)	(II)
Crystal data		
Chemical formula	C_12_H_12_N_6_O_3_S·H_2_O	C_12_H_12_ClN_5_OS
*M*_r_	338.35	309.78
Crystal system, space group	Orthorhombic, *P* *n* *a*2_1_	Triclinic, *P* 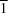
Temperature (K)	293	293
*a*, *b*, *c* (Å)	7.2326 (1), 14.3442 (2), 14.0940 (3)	7.2528 (2), 7.6249 (3), 13.0649 (4)
α, β, γ (°)	90, 90, 90	91.410 (2), 105.924 (2), 94.647 (2)
*V* (Å^3^)	1462.19 (4)	691.68 (4)
*Z*	4	2
Radiation type	Mo *K*α	Mo *K*α
μ (mm^−1^)	0.25	0.43
Crystal size (mm)	0.30 × 0.25 × 0.20	0.30 × 0.20 × 0.15

Data collection		
Diffractometer	Bruker SMART APEXII area-detector	Bruker SMART APEXII area-detector
Absorption correction	Multi-scan (*SADABS*; Bruker, 2008 ▸)	Multi-scan (*SADABS*; Bruker, 2008 ▸)
*T*_min_, *T*_max_	0.785, 0.845	0.785, 0.845
No. of measured, independent and observed [*I* > 2σ(*I*)] reflections	7912, 3265, 3034	10154, 2822, 2519
*R*_int_	0.021	0.022
(sin θ/λ)_max_ (Å^−1^)	0.667	0.626

Refinement		
*R*[*F* ^2^ > 2σ(*F* ^2^)], *wR*(*F* ^2^), *S*	0.027, 0.069, 1.04	0.035, 0.099, 1.04
No. of reflections	3265	2822
No. of parameters	229	201
No. of restraints	1	0
H-atom treatment	H atoms treated by a mixture of independent and constrained refinement	H atoms treated by a mixture of independent and constrained refinement
Δρ_max_, Δρ_min_ (e Å^−3^)	0.16, −0.17	0.50, −0.50
Absolute structure	Flack x determined using 1217 quotients [(*I* ^+^)−(*I* ^−^)]/[(*I* ^+^)+(*I* ^−^)] (Parsons *et al.*, 2013 ▸)	–
Absolute structure parameter	0.07 (3)	–

Computer programs: *APEX2* (Bruker, 2008 ▸), *SAINT* (Bruker, 2008 ▸), *SHELXS97* (Sheldrick, 2008 ▸), *Mercury* (Macrae *et al.*, 2008 ▸) and *PLATON* (Spek, 2009 ▸), *SHELXL2014* (Sheldrick, 2015 ▸) and *PLATON* (Spek, 2009 ▸).

## supplementary crystallographic information

### (I) 2-[(4,6-Diaminopyrimidin-2-yl)sulfanyl]-N-(3-nitrophenyl)acetamide monohydrate Crystal data

**Table:**

C_12_H_12_N_6_O_3_S·H_2_O	*D*_x_ = 1.537 Mg m^−^^3^
*M**_r_* = 338.35	Mo *K*α radiation, λ = 0.71073 Å
Orthorhombic, *P**n**a*2_1_	Cell parameters from 3265 reflections
*a* = 7.2326 (1) Å	θ = 2.0–28.3°
*b* = 14.3442 (2) Å	µ = 0.25 mm^−^^1^
*c* = 14.0940 (3) Å	*T* = 293 K
*V* = 1462.19 (4) Å^3^	Block, colourless
*Z* = 4	0.30 × 0.25 × 0.20 mm
*F*(000) = 704	

### (I) 2-[(4,6-Diaminopyrimidin-2-yl)sulfanyl]-N-(3-nitrophenyl)acetamide monohydrate Data collection

**Table:**

Bruker SMART APEXII area-detector diffractometer	3034 reflections with *I* > 2σ(*I*)
ω and φ scans	*R*_int_ = 0.021
Absorption correction: multi-scan (*SADABS*; Bruker, 2008)	θ_max_ = 28.3°, θ_min_ = 2.0°
*T*_min_ = 0.785, *T*_max_ = 0.845	*h* = −5→9
7912 measured reflections	*k* = −18→19
3265 independent reflections	*l* = −15→18

### (I) 2-[(4,6-Diaminopyrimidin-2-yl)sulfanyl]-N-(3-nitrophenyl)acetamide monohydrate Refinement

**Table:**

Refinement on *F*^2^	Hydrogen site location: mixed
Least-squares matrix: full	H atoms treated by a mixture of independent and constrained refinement
*R*[*F*^2^ > 2σ(*F*^2^)] = 0.027	*w* = 1/[σ^2^(*F*_o_^2^) + (0.0396*P*)^2^ + 0.1263*P*] where *P* = (*F*_o_^2^ + 2*F*_c_^2^)/3
*wR*(*F*^2^) = 0.069	(Δ/σ)_max_ = 0.001
*S* = 1.04	Δρ_max_ = 0.16 e Å^−^^3^
3265 reflections	Δρ_min_ = −0.17 e Å^−^^3^
229 parameters	Extinction correction: SHELXL, Fc^*^=kFc[1+0.001xFc^2^λ^3^/sin(2θ)]^-1/4^
1 restraint	Extinction coefficient: 0.0039 (10)
Primary atom site location: structure-invariant direct methods	Absolute structure: Flack x determined using 1217 quotients [(*I*^+^)-(*I*^-^)]/[(*I*^+^)+(*I*^-^)] (Parsons *et al.*, 2013)
Secondary atom site location: difference Fourier map	Absolute structure parameter: 0.07 (3)

### (I) 2-[(4,6-Diaminopyrimidin-2-yl)sulfanyl]-N-(3-nitrophenyl)acetamide monohydrate Special details

**Table:**

Geometry. All esds (except the esd in the dihedral angle between two l.s. planes) are estimated using the full covariance matrix. The cell esds are taken into account individually in the estimation of esds in distances, angles and torsion angles; correlations between esds in cell parameters are only used when they are defined by crystal symmetry. An approximate (isotropic) treatment of cell esds is used for estimating esds involving l.s. planes.

### (I) 2-[(4,6-Diaminopyrimidin-2-yl)sulfanyl]-N-(3-nitrophenyl)acetamide monohydrate Fractional atomic coordinates and isotropic or equivalent isotropic displacement parameters (Å^2^)

**Table:**

	*x*	*y*	*z*	*U*_iso_*/*U*_eq_	
S1	0.12955 (8)	0.37215 (3)	0.66439 (4)	0.03810 (14)	
O1	0.2112 (3)	0.32497 (11)	0.42274 (13)	0.0555 (5)	
O2	0.4515 (3)	0.40381 (12)	0.13791 (14)	0.0570 (5)	
O3	0.4586 (3)	0.53670 (14)	0.06709 (14)	0.0602 (5)	
N1	−0.0426 (3)	0.69929 (14)	0.58998 (15)	0.0483 (5)	
H1A	−0.112 (3)	0.6759 (19)	0.550 (2)	0.039 (7)*	
H1B	−0.049 (4)	0.7564 (18)	0.601 (2)	0.048 (7)*	
N2	0.2912 (3)	0.62828 (15)	0.87403 (16)	0.0473 (5)	
H2A	0.343 (4)	0.587 (2)	0.902 (2)	0.052 (9)*	
H2B	0.303 (4)	0.683 (2)	0.891 (2)	0.058 (8)*	
N3	0.2016 (2)	0.51355 (12)	0.77054 (12)	0.0346 (4)	
N4	0.0368 (2)	0.54948 (11)	0.62840 (12)	0.0327 (4)	
N5	0.1324 (3)	0.47612 (12)	0.44843 (13)	0.0361 (4)	
H5	0.081 (4)	0.5115 (19)	0.490 (2)	0.050 (7)*	
N6	0.4308 (3)	0.48792 (13)	0.13678 (13)	0.0404 (4)	
C1	0.0378 (3)	0.64158 (12)	0.65303 (16)	0.0351 (4)	
C2	0.1209 (3)	0.67214 (15)	0.73550 (15)	0.0386 (5)	
H2	0.1206	0.7349	0.7522	0.046*	
C3	0.2048 (3)	0.60567 (14)	0.79264 (17)	0.0359 (4)	
C4	0.1191 (3)	0.49297 (13)	0.68900 (14)	0.0318 (4)	
C5	0.0093 (3)	0.36034 (14)	0.55277 (16)	0.0369 (5)	
H5A	−0.0345	0.2967	0.5466	0.044*	
H5B	−0.0979	0.4010	0.5532	0.044*	
C6	0.1278 (3)	0.38380 (14)	0.46763 (15)	0.0350 (4)	
C7	0.2255 (3)	0.52300 (13)	0.37571 (15)	0.0327 (4)	
C8	0.2440 (4)	0.61934 (14)	0.38488 (18)	0.0418 (5)	
H8	0.2021	0.6488	0.4396	0.050*	
C9	0.3239 (4)	0.67138 (15)	0.31366 (19)	0.0471 (6)	
H9	0.3368	0.7355	0.3211	0.057*	
C10	0.3850 (3)	0.62925 (15)	0.23135 (18)	0.0418 (5)	
H10	0.4364	0.6640	0.1823	0.050*	
C11	0.3670 (3)	0.53390 (15)	0.22449 (15)	0.0339 (4)	
C12	0.2907 (3)	0.47907 (13)	0.29473 (15)	0.0331 (4)	
H12	0.2832	0.4147	0.2880	0.040*	
O1W	0.7494 (3)	0.61566 (15)	0.42859 (15)	0.0615 (5)	
H1WA	0.7637	0.5885	0.3748	0.092*	
H1WB	0.6377	0.6003	0.4521	0.092*	

### (I) 2-[(4,6-Diaminopyrimidin-2-yl)sulfanyl]-N-(3-nitrophenyl)acetamide monohydrate Atomic displacement parameters (Å^2^)

**Table:**

	*U*^11^	*U*^22^	*U*^33^	*U*^12^	*U*^13^	*U*^23^
S1	0.0581 (3)	0.0259 (2)	0.0302 (2)	−0.00098 (19)	−0.0040 (2)	0.0025 (2)
O1	0.0851 (13)	0.0319 (8)	0.0494 (11)	0.0075 (8)	0.0205 (9)	−0.0010 (7)
O2	0.0763 (12)	0.0476 (9)	0.0470 (11)	0.0082 (9)	0.0072 (9)	−0.0122 (8)
O3	0.0765 (12)	0.0711 (12)	0.0328 (9)	−0.0071 (10)	0.0107 (9)	0.0022 (9)
N1	0.0692 (15)	0.0309 (9)	0.0448 (12)	0.0072 (9)	−0.0120 (11)	−0.0024 (8)
N2	0.0644 (14)	0.0385 (11)	0.0390 (12)	−0.0024 (10)	−0.0091 (10)	−0.0079 (9)
N3	0.0424 (9)	0.0331 (8)	0.0283 (9)	−0.0021 (7)	0.0003 (7)	−0.0017 (7)
N4	0.0427 (9)	0.0278 (7)	0.0278 (8)	−0.0008 (7)	0.0018 (7)	−0.0007 (6)
N5	0.0507 (11)	0.0288 (8)	0.0287 (9)	0.0018 (7)	0.0073 (8)	−0.0020 (7)
N6	0.0394 (9)	0.0484 (10)	0.0336 (10)	−0.0015 (8)	−0.0007 (8)	−0.0030 (8)
C1	0.0408 (10)	0.0297 (8)	0.0349 (11)	0.0008 (7)	0.0066 (9)	−0.0008 (8)
C2	0.0517 (13)	0.0281 (9)	0.0360 (11)	−0.0036 (9)	0.0036 (10)	−0.0058 (8)
C3	0.0396 (10)	0.0362 (9)	0.0320 (11)	−0.0049 (9)	0.0062 (8)	−0.0038 (8)
C4	0.0378 (10)	0.0285 (9)	0.0290 (11)	−0.0033 (8)	0.0059 (8)	−0.0007 (7)
C5	0.0467 (11)	0.0319 (10)	0.0322 (11)	−0.0077 (8)	−0.0008 (9)	−0.0009 (8)
C6	0.0446 (12)	0.0303 (9)	0.0302 (11)	−0.0013 (8)	−0.0024 (9)	−0.0007 (7)
C7	0.0395 (11)	0.0295 (9)	0.0292 (10)	−0.0005 (8)	−0.0003 (8)	0.0008 (8)
C8	0.0561 (13)	0.0317 (10)	0.0376 (12)	0.0005 (9)	0.0074 (10)	−0.0035 (9)
C9	0.0664 (15)	0.0280 (9)	0.0469 (13)	−0.0015 (10)	0.0084 (12)	0.0025 (9)
C10	0.0500 (13)	0.0350 (11)	0.0404 (13)	−0.0008 (9)	0.0078 (11)	0.0076 (9)
C11	0.0360 (10)	0.0369 (10)	0.0288 (10)	0.0026 (9)	−0.0001 (8)	−0.0002 (8)
C12	0.0380 (10)	0.0301 (8)	0.0311 (10)	−0.0006 (8)	−0.0011 (8)	−0.0003 (8)
O1W	0.0706 (12)	0.0695 (12)	0.0444 (11)	−0.0050 (9)	−0.0003 (10)	−0.0118 (9)

### (I) 2-[(4,6-Diaminopyrimidin-2-yl)sulfanyl]-N-(3-nitrophenyl)acetamide monohydrate Geometric parameters (Å, º)

**Table:**

S1—C4	1.769 (2)	C1—C2	1.380 (3)
S1—C5	1.805 (2)	C2—C3	1.388 (3)
O1—C6	1.215 (3)	C2—H2	0.9300
O2—N6	1.216 (2)	C5—C6	1.512 (3)
O3—N6	1.223 (3)	C5—H5A	0.9700
N1—C1	1.347 (3)	C5—H5B	0.9700
N1—H1A	0.83 (3)	C7—C12	1.386 (3)
N1—H1B	0.84 (3)	C7—C8	1.394 (3)
N2—C3	1.346 (3)	C8—C9	1.378 (3)
N2—H2A	0.80 (3)	C8—H8	0.9300
N2—H2B	0.83 (3)	C9—C10	1.381 (3)
N3—C4	1.328 (3)	C9—H9	0.9300
N3—C3	1.358 (3)	C10—C11	1.377 (3)
N4—C4	1.319 (3)	C10—H10	0.9300
N4—C1	1.366 (2)	C11—C12	1.380 (3)
N5—C6	1.352 (3)	C12—H12	0.9300
N5—C7	1.398 (3)	O1W—H1WA	0.8582
N5—H5	0.86 (3)	O1W—H1WB	0.9004
N6—C11	1.475 (3)		
			
C4—S1—C5	104.01 (9)	C6—C5—H5A	108.9
C1—N1—H1A	117.8 (18)	S1—C5—H5A	108.9
C1—N1—H1B	120 (2)	C6—C5—H5B	108.9
H1A—N1—H1B	120 (3)	S1—C5—H5B	108.9
C3—N2—H2A	117 (2)	H5A—C5—H5B	107.7
C3—N2—H2B	121 (2)	O1—C6—N5	124.3 (2)
H2A—N2—H2B	121 (3)	O1—C6—C5	122.68 (19)
C4—N3—C3	114.97 (18)	N5—C6—C5	113.01 (18)
C4—N4—C1	115.30 (18)	C12—C7—C8	119.6 (2)
C6—N5—C7	129.02 (18)	C12—C7—N5	123.27 (17)
C6—N5—H5	115.5 (19)	C8—C7—N5	117.05 (19)
C7—N5—H5	115.1 (19)	C9—C8—C7	120.6 (2)
O2—N6—O3	123.9 (2)	C9—C8—H8	119.7
O2—N6—C11	118.11 (18)	C7—C8—H8	119.7
O3—N6—C11	117.96 (18)	C8—C9—C10	120.6 (2)
N1—C1—N4	115.1 (2)	C8—C9—H9	119.7
N1—C1—C2	123.28 (19)	C10—C9—H9	119.7
N4—C1—C2	121.57 (19)	C11—C10—C9	117.6 (2)
C1—C2—C3	117.44 (19)	C11—C10—H10	121.2
C1—C2—H2	121.3	C9—C10—H10	121.2
C3—C2—H2	121.3	C10—C11—C12	123.6 (2)
N2—C3—N3	116.0 (2)	C10—C11—N6	118.26 (19)
N2—C3—C2	122.1 (2)	C12—C11—N6	118.14 (18)
N3—C3—C2	121.9 (2)	C11—C12—C7	117.89 (17)
N4—C4—N3	128.79 (18)	C11—C12—H12	121.1
N4—C4—S1	119.62 (15)	C7—C12—H12	121.1
N3—C4—S1	111.59 (15)	H1WA—O1W—H1WB	108.8
C6—C5—S1	113.43 (15)		
			
C4—N4—C1—N1	178.57 (19)	S1—C5—C6—N5	−84.2 (2)
C4—N4—C1—C2	0.5 (3)	C6—N5—C7—C12	18.1 (3)
N1—C1—C2—C3	−177.6 (2)	C6—N5—C7—C8	−164.9 (2)
N4—C1—C2—C3	0.3 (3)	C12—C7—C8—C9	1.0 (4)
C4—N3—C3—N2	−178.7 (2)	N5—C7—C8—C9	−176.0 (2)
C4—N3—C3—C2	2.5 (3)	C7—C8—C9—C10	0.8 (4)
C1—C2—C3—N2	179.3 (2)	C8—C9—C10—C11	−1.5 (4)
C1—C2—C3—N3	−1.9 (3)	C9—C10—C11—C12	0.5 (3)
C1—N4—C4—N3	0.3 (3)	C9—C10—C11—N6	179.7 (2)
C1—N4—C4—S1	−179.23 (14)	O2—N6—C11—C10	164.8 (2)
C3—N3—C4—N4	−1.7 (3)	O3—N6—C11—C10	−15.6 (3)
C3—N3—C4—S1	177.82 (16)	O2—N6—C11—C12	−15.9 (3)
C5—S1—C4—N4	−0.85 (19)	O3—N6—C11—C12	163.6 (2)
C5—S1—C4—N3	179.56 (14)	C10—C11—C12—C7	1.3 (3)
C4—S1—C5—C6	81.31 (16)	N6—C11—C12—C7	−177.94 (17)
C7—N5—C6—O1	1.4 (4)	C8—C7—C12—C11	−2.0 (3)
C7—N5—C6—C5	−179.6 (2)	N5—C7—C12—C11	174.86 (19)
S1—C5—C6—O1	94.8 (2)		

### (I) 2-[(4,6-Diaminopyrimidin-2-yl)sulfanyl]-N-(3-nitrophenyl)acetamide monohydrate Hydrogen-bond geometry (Å, º)

**Table:**

*D*—H···*A*	*D*—H	H···*A*	*D*···*A*	*D*—H···*A*
N5—H5···N4	0.86 (3)	2.05 (3)	2.832 (3)	151 (3)
C12—H12···O1	0.93	2.35	2.911 (3)	118
N1—H1*A*···O1*W*^i^	0.83 (3)	2.16 (3)	2.979 (3)	170 (2)
N1—H1*B*···O2^ii^	0.84 (3)	2.29 (3)	3.082 (3)	159 (3)
N2—H2*A*···O3^iii^	0.80 (3)	2.58 (3)	3.255 (3)	143 (3)
N2—H2*B*···O1^ii^	0.83 (3)	2.09 (3)	2.904 (3)	170 (3)
O1*W*—H1*WA*···N3^iv^	0.86	2.09	2.919 (3)	162
O1*W*—H1*WB*···O3^v^	0.90	2.64	3.294 (3)	130

Symmetry codes: (i) *x*−1, *y*, *z*; (ii) −*x*+1/2, *y*+1/2, *z*+1/2; (iii) *x*, *y*, *z*+1; (iv) −*x*+1, −*y*+1, *z*−1/2; (v) −*x*+1, −*y*+1, *z*+1/2.

### (II) N-(2-Chlorophenyl)-2-[(4,6-diaminopyrimidin-2-yl)sulfanyl]acetamide Crystal data

**Table:**

C_12_H_12_ClN_5_OS	*Z* = 2
*M**_r_* = 309.78	*F*(000) = 320
Triclinic, *P*1	*D*_x_ = 1.487 Mg m^−^^3^
*a* = 7.2528 (2) Å	Mo *K*α radiation, λ = 0.71073 Å
*b* = 7.6249 (3) Å	Cell parameters from 2822 reflections
*c* = 13.0649 (4) Å	θ = 1.6–26.4°
α = 91.410 (2)°	µ = 0.43 mm^−^^1^
β = 105.924 (2)°	*T* = 293 K
γ = 94.647 (2)°	Block, colourless
*V* = 691.68 (4) Å^3^	0.30 × 0.20 × 0.15 mm

### (II) N-(2-Chlorophenyl)-2-[(4,6-diaminopyrimidin-2-yl)sulfanyl]acetamide Data collection

**Table:**

Bruker SMART APEXII area-detector diffractometer	2519 reflections with *I* > 2σ(*I*)
ω and φ scans	*R*_int_ = 0.022
Absorption correction: multi-scan (*SADABS*; Bruker, 2008)	θ_max_ = 26.4°, θ_min_ = 1.6°
*T*_min_ = 0.785, *T*_max_ = 0.845	*h* = −9→8
10154 measured reflections	*k* = −9→9
2822 independent reflections	*l* = −16→16

### (II) N-(2-Chlorophenyl)-2-[(4,6-diaminopyrimidin-2-yl)sulfanyl]acetamide Refinement

**Table:**

Refinement on *F*^2^	Primary atom site location: structure-invariant direct methods
Least-squares matrix: full	Secondary atom site location: difference Fourier map
*R*[*F*^2^ > 2σ(*F*^2^)] = 0.035	Hydrogen site location: mixed
*wR*(*F*^2^) = 0.099	H atoms treated by a mixture of independent and constrained refinement
*S* = 1.04	*w* = 1/[σ^2^(*F*_o_^2^) + (0.0515*P*)^2^ + 0.2692*P*] where *P* = (*F*_o_^2^ + 2*F*_c_^2^)/3
2822 reflections	(Δ/σ)_max_ < 0.001
201 parameters	Δρ_max_ = 0.50 e Å^−^^3^
0 restraints	Δρ_min_ = −0.50 e Å^−^^3^

### (II) N-(2-Chlorophenyl)-2-[(4,6-diaminopyrimidin-2-yl)sulfanyl]acetamide Special details

**Table:**

Geometry. All esds (except the esd in the dihedral angle between two l.s. planes) are estimated using the full covariance matrix. The cell esds are taken into account individually in the estimation of esds in distances, angles and torsion angles; correlations between esds in cell parameters are only used when they are defined by crystal symmetry. An approximate (isotropic) treatment of cell esds is used for estimating esds involving l.s. planes.

### (II) N-(2-Chlorophenyl)-2-[(4,6-diaminopyrimidin-2-yl)sulfanyl]acetamide Fractional atomic coordinates and isotropic or equivalent isotropic displacement parameters (Å^2^)

**Table:**

	*x*	*y*	*z*	*U*_iso_*/*U*_eq_	
Cl1	−0.32045 (7)	0.83502 (8)	0.11080 (5)	0.06488 (18)	
S1	0.22688 (6)	0.44103 (6)	0.41282 (3)	0.04714 (15)	
O1	0.35760 (17)	0.81194 (17)	0.26781 (10)	0.0503 (3)	
N1	−0.2505 (3)	−0.0531 (2)	0.40354 (14)	0.0541 (4)	
H1A	−0.163 (3)	−0.091 (3)	0.4537 (18)	0.051 (6)*	
H1B	−0.361 (3)	−0.101 (3)	0.3829 (18)	0.058 (6)*	
N2	−0.4671 (2)	0.4665 (2)	0.23211 (16)	0.0576 (5)	
H2A	−0.437 (3)	0.572 (3)	0.2321 (18)	0.062 (7)*	
H2B	−0.584 (4)	0.428 (3)	0.2221 (19)	0.070 (7)*	
N3	−0.03937 (19)	0.19020 (19)	0.40540 (10)	0.0396 (3)	
N4	−0.14840 (18)	0.44884 (18)	0.31711 (10)	0.0371 (3)	
N5	0.0527 (2)	0.6847 (2)	0.20471 (11)	0.0416 (3)	
H5	−0.040 (3)	0.631 (3)	0.2233 (15)	0.045 (5)*	
C1	−0.2221 (2)	0.1113 (2)	0.37325 (12)	0.0383 (3)	
C2	−0.3723 (2)	0.1992 (2)	0.31353 (13)	0.0403 (4)	
H2	−0.4970	0.1447	0.2910	0.048*	
C3	−0.3310 (2)	0.3694 (2)	0.28860 (13)	0.0378 (3)	
C4	−0.0176 (2)	0.3525 (2)	0.37342 (11)	0.0353 (3)	
C5	0.2110 (3)	0.6742 (2)	0.39173 (13)	0.0453 (4)	
H5A	0.0924	0.7068	0.4045	0.054*	
H5B	0.3169	0.7396	0.4442	0.054*	
C6	0.2155 (2)	0.7302 (2)	0.28193 (12)	0.0359 (3)	
C7	0.0187 (2)	0.7245 (2)	0.09613 (12)	0.0375 (3)	
C8	−0.1521 (2)	0.7917 (2)	0.04306 (14)	0.0428 (4)	
C9	−0.1897 (3)	0.8314 (3)	−0.06301 (15)	0.0545 (5)	
H9	−0.3048	0.8763	−0.0976	0.065*	
C10	−0.0557 (4)	0.8039 (3)	−0.11667 (15)	0.0603 (6)	
H10	−0.0795	0.8312	−0.1879	0.072*	
C11	0.1148 (3)	0.7360 (3)	−0.06546 (15)	0.0556 (5)	
H11	0.2048	0.7168	−0.1024	0.067*	
C12	0.1516 (3)	0.6965 (2)	0.04065 (14)	0.0454 (4)	
H12	0.2665	0.6508	0.0748	0.055*	

### (II) N-(2-Chlorophenyl)-2-[(4,6-diaminopyrimidin-2-yl)sulfanyl]acetamide Atomic displacement parameters (Å^2^)

**Table:**

	*U*^11^	*U*^22^	*U*^33^	*U*^12^	*U*^13^	*U*^23^
Cl1	0.0473 (3)	0.0730 (4)	0.0803 (4)	0.0106 (2)	0.0244 (2)	0.0255 (3)
S1	0.0309 (2)	0.0609 (3)	0.0435 (2)	−0.00578 (18)	0.00128 (17)	0.0193 (2)
O1	0.0404 (6)	0.0548 (7)	0.0492 (7)	−0.0158 (5)	0.0072 (5)	0.0071 (6)
N1	0.0440 (9)	0.0474 (9)	0.0579 (10)	−0.0079 (7)	−0.0061 (8)	0.0187 (7)
N2	0.0319 (8)	0.0501 (10)	0.0858 (13)	0.0021 (7)	0.0066 (8)	0.0271 (9)
N3	0.0339 (7)	0.0466 (8)	0.0349 (7)	−0.0005 (6)	0.0043 (5)	0.0105 (6)
N4	0.0318 (6)	0.0431 (7)	0.0360 (7)	−0.0010 (5)	0.0094 (5)	0.0082 (5)
N5	0.0339 (7)	0.0515 (8)	0.0360 (7)	−0.0097 (6)	0.0072 (6)	0.0099 (6)
C1	0.0388 (8)	0.0418 (8)	0.0307 (7)	−0.0015 (7)	0.0052 (6)	0.0049 (6)
C2	0.0314 (8)	0.0458 (9)	0.0390 (8)	−0.0034 (6)	0.0037 (6)	0.0068 (7)
C3	0.0322 (8)	0.0447 (9)	0.0364 (8)	0.0017 (6)	0.0097 (6)	0.0070 (6)
C4	0.0315 (7)	0.0469 (9)	0.0262 (7)	−0.0018 (6)	0.0073 (6)	0.0049 (6)
C5	0.0447 (9)	0.0511 (10)	0.0343 (8)	−0.0120 (7)	0.0062 (7)	−0.0025 (7)
C6	0.0355 (8)	0.0324 (7)	0.0376 (8)	−0.0031 (6)	0.0082 (6)	0.0013 (6)
C7	0.0398 (8)	0.0337 (8)	0.0350 (8)	−0.0089 (6)	0.0069 (6)	0.0043 (6)
C8	0.0405 (9)	0.0384 (8)	0.0446 (9)	−0.0071 (7)	0.0061 (7)	0.0053 (7)
C9	0.0582 (11)	0.0472 (10)	0.0447 (10)	−0.0083 (8)	−0.0049 (8)	0.0103 (8)
C10	0.0873 (15)	0.0526 (11)	0.0325 (9)	−0.0121 (10)	0.0073 (9)	0.0031 (8)
C11	0.0761 (14)	0.0496 (10)	0.0447 (10)	−0.0064 (9)	0.0269 (10)	−0.0024 (8)
C12	0.0486 (10)	0.0421 (9)	0.0456 (9)	−0.0015 (7)	0.0145 (8)	0.0029 (7)

### (II) N-(2-Chlorophenyl)-2-[(4,6-diaminopyrimidin-2-yl)sulfanyl]acetamide Geometric parameters (Å, º)

**Table:**

Cl1—C8	1.7397 (19)	C1—C2	1.386 (2)
S1—C4	1.7753 (15)	C2—C3	1.375 (2)
S1—C5	1.8135 (19)	C2—H2	0.9300
O1—C6	1.2207 (19)	C5—C6	1.515 (2)
N1—C1	1.340 (2)	C5—H5A	0.9700
N1—H1A	0.85 (2)	C5—H5B	0.9700
N1—H1B	0.83 (2)	C7—C12	1.382 (2)
N2—C3	1.346 (2)	C7—C8	1.388 (2)
N2—H2A	0.81 (3)	C8—C9	1.383 (3)
N2—H2B	0.85 (3)	C9—C10	1.371 (3)
N3—C4	1.327 (2)	C9—H9	0.9300
N3—C1	1.359 (2)	C10—C11	1.382 (3)
N4—C4	1.318 (2)	C10—H10	0.9300
N4—C3	1.360 (2)	C11—C12	1.384 (3)
N5—C6	1.340 (2)	C11—H11	0.9300
N5—C7	1.417 (2)	C12—H12	0.9300
N5—H5	0.85 (2)		
			
C4—S1—C5	103.22 (8)	S1—C5—H5A	108.5
C1—N1—H1A	118.4 (15)	C6—C5—H5B	108.5
C1—N1—H1B	116.7 (16)	S1—C5—H5B	108.5
H1A—N1—H1B	123 (2)	H5A—C5—H5B	107.5
C3—N2—H2A	116.6 (17)	O1—C6—N5	124.20 (15)
C3—N2—H2B	118.0 (17)	O1—C6—C5	121.24 (15)
H2A—N2—H2B	120 (2)	N5—C6—C5	114.56 (14)
C4—N3—C1	115.11 (13)	C12—C7—C8	118.68 (15)
C4—N4—C3	114.72 (13)	C12—C7—N5	121.45 (15)
C6—N5—C7	125.63 (14)	C8—C7—N5	119.87 (15)
C6—N5—H5	116.8 (13)	C9—C8—C7	121.22 (18)
C7—N5—H5	117.5 (13)	C9—C8—Cl1	118.54 (15)
N1—C1—N3	117.08 (15)	C7—C8—Cl1	120.20 (13)
N1—C1—C2	121.83 (15)	C10—C9—C8	119.36 (19)
N3—C1—C2	121.08 (14)	C10—C9—H9	120.3
C3—C2—C1	117.94 (14)	C8—C9—H9	120.3
C3—C2—H2	121.0	C9—C10—C11	120.31 (17)
C1—C2—H2	121.0	C9—C10—H10	119.8
N2—C3—N4	115.59 (15)	C11—C10—H10	119.8
N2—C3—C2	122.48 (15)	C10—C11—C12	120.09 (19)
N4—C3—C2	121.90 (15)	C10—C11—H11	120.0
N4—C4—N3	129.18 (14)	C12—C11—H11	120.0
N4—C4—S1	118.71 (12)	C7—C12—C11	120.34 (18)
N3—C4—S1	112.10 (11)	C7—C12—H12	119.8
C6—C5—S1	115.29 (12)	C11—C12—H12	119.8
C6—C5—H5A	108.5		
			
C4—N3—C1—N1	179.80 (16)	C7—N5—C6—C5	178.60 (16)
C4—N3—C1—C2	−1.4 (2)	S1—C5—C6—O1	−106.66 (16)
N1—C1—C2—C3	178.13 (17)	S1—C5—C6—N5	74.10 (18)
N3—C1—C2—C3	−0.6 (3)	C6—N5—C7—C12	47.4 (2)
C4—N4—C3—N2	179.24 (16)	C6—N5—C7—C8	−133.27 (18)
C4—N4—C3—C2	−2.5 (2)	C12—C7—C8—C9	−0.5 (2)
C1—C2—C3—N2	−179.19 (18)	N5—C7—C8—C9	−179.80 (15)
C1—C2—C3—N4	2.7 (3)	C12—C7—C8—Cl1	−178.14 (12)
C3—N4—C4—N3	0.3 (2)	N5—C7—C8—Cl1	2.5 (2)
C3—N4—C4—S1	178.72 (11)	C7—C8—C9—C10	0.0 (3)
C1—N3—C4—N4	1.7 (2)	Cl1—C8—C9—C10	177.71 (14)
C1—N3—C4—S1	−176.86 (11)	C8—C9—C10—C11	0.5 (3)
C5—S1—C4—N4	15.56 (15)	C9—C10—C11—C12	−0.5 (3)
C5—S1—C4—N3	−165.73 (12)	C8—C7—C12—C11	0.5 (2)
C4—S1—C5—C6	−89.86 (13)	N5—C7—C12—C11	179.79 (15)
C7—N5—C6—O1	−0.6 (3)	C10—C11—C12—C7	0.0 (3)

### (II) N-(2-Chlorophenyl)-2-[(4,6-diaminopyrimidin-2-yl)sulfanyl]acetamide Hydrogen-bond geometry (Å, º)

**Table:**

*D*—H···*A*	*D*—H	H···*A*	*D*···*A*	*D*—H···*A*
N5—H5···N4	0.85 (2)	2.12 (2)	2.898 (2)	152 (2)
N2—H2*A*···Cl1	0.81 (3)	2.81 (2)	3.493 (2)	143 (2)
N1—H1*A*···N3^i^	0.85 (2)	2.21 (2)	3.058 (2)	174 (2)
N1—H1*B*···O1^ii^	0.83 (2)	2.21 (2)	2.992 (2)	157 (2)
N2—H2*A*···O1^iii^	0.81 (3)	2.56 (2)	3.095 (2)	124 (2)
C2—H2···O1^ii^	0.93	2.64	3.353 (2)	134

Symmetry codes: (i) −*x*, −*y*, −*z*+1; (ii) *x*−1, *y*−1, *z*; (iii) *x*−1, *y*, *z*.
